# Development and Validation of the Predictive Model for Esophageal Squamous Cell Carcinoma Differentiation Degree

**DOI:** 10.3389/fgene.2020.595638

**Published:** 2020-10-23

**Authors:** Yanfeng Wang, Yuli Yang, Junwei Sun, Lidong Wang, Xin Song, Xueke Zhao

**Affiliations:** ^1^Henan Key Lab of Information-Based Electrical Appliances, Zhengzhou University of Light Industry, Zhengzhou, China; ^2^State Key Laboratory of Esophageal Cancer Prevention & Treatment and Henan Key Laboratory for Esophageal Cancer Research of The First Affiliated Hospital, Zhengzhou University, Zhengzhou, China

**Keywords:** ESCC, degree of differentiation, prediction model, clustering algorithm, ABC-SVM, ROC, ReliefF algorithm

## Abstract

The diagnosis of the degree of differentiation of tumor cells can help physicians to make timely detection and take appropriate treatment for the patient's condition. In this study, the original dataset is clustered into two independent types by the Kohonen clustering algorithm. One type is used as the development sets to find correlation indicators and establish predictive models of differentiation, while the other type is used as the validation sets to test the correlation indicators and models. In the development sets, thirteen indicators significantly associated with the degree of differentiation of esophageal squamous cell carcinoma are found by the Kohonen clustering algorithm. Thirteen relevant indicators are used as input features and the degree of tumor differentiations is used as output. Ten classification algorithms are used to predict the differentiation of esophageal squamous cell carcinoma. Artificial bee colony-support vector machine (ABC-SVM) predicts better than the other nine algorithms, with an average accuracy of 81.5% for the 10-fold cross-validation. Based on logistic regression and ReliefF algorithm, five models with the greater merit for the degree of differentiation are found in the development sets. The AUC values of the five models are 0.672, 0.628, 0.630, 0.628, and 0.608 (*P* < 0.05). The AUC values of the five models in the validation sets are 0.753, 0.728, 0.744, 0.776, and 0.868 (*P* < 0.0001). The predicted values of the five models are constructed as the input features of ABC-SVM. The accuracy of the 10-fold cross-validation reached 82.0 and 86.5% in the development sets and the validation sets, respectively.

## 1. Introduction

Esophageal squamous cell carcinoma (ESCC) is one of the most common malignant tumors in China, which has a high mortality rate (McCormack et al., 2017; Domingues et al., 2019; Hou et al., 2019). The degree of tumor cell differentiation of esophageal squamous cell carcinoma is an important reference information in cancer diagnosis and treatment. High differentiation means that the tumor cells are more similar to normal cells, the tumor is less malignant and less likely to metastasize. It is less sensitive to radiotherapy and chemotherapy and has better prognosis. The difference between low-differentiated cells and normal cells is very big, and the malignancy of tumor is relatively high. It is easy to metastasize in the clinical process, and it is more sensitive to radiotherapy and chemotherapy, so the prognosis is poor. As long as early detection and timely treatment can be done, the metastatic speed of tumor can be slowed down through integrated treatment of traditional Chinese and western medicine, and achieve better clinical efficacy (Cong et al., 2019).

Cancer cells have the characteristic of differentiating into normal cells (Tamaoki et al., 2018). In medicine, this feature is used by doctors to determine the degree of differentiation of tumor cells. After the patient's biopsy pathology, the malignancy and differentiation of the tumor are confirmed, by observing the characteristic state of tumor cells under a microscope. The traditional method of determining the degree of differentiation is complicated and needs to rely on human experience to make decisions (Maehara et al., 2018; Jadcherla et al., 2019). In this paper, we aim to develop a new model to predict the degree of differentiation of esophageal cancer patients based on blood indicators and tumor size parameters. The prediction model can better predict the degree of esophageal cancer tumor differentiation, which can assist professional physicians in making decisions and improve the clinical treatment effect.

The original dataset is clustered into two distinct datasets by the Kohonen algorithm. The first dataset is used to develop the prediction model for the degree of esophageal squamous cell carcinoma differentiation and the second dataset is used to validate the prediction model. First, in the development sets, the Kohonen clustering algorithm is used to cluster multiple indicators significantly associated with esophageal squamous cell carcinoma. Thirteen indicators significantly associated with the degree of esophageal squamous cell carcinoma differentiation are found. Based on these 13 indicators, 10 classification algorithms are used to predict the degree of differentiation. The results show that ABC-SVM predicts better than the other nine algorithms, with an average accuracy of 81.5% for the 10-fold cross-validation. Then, logistic regression and ReliefF algorithm are used to find five models that have greater predictive value for the degree of esophageal cancer differentiation. The AUC values of the five models in the development sets are 0.672, 0.628, 0.630, 0.628, and 0.608, with *P*-values less than 0.05. The AUC values of the five models in the validation sets are 0.753, 0.728, 0.744, 0.776, and 0.868, with *P*-values less than 0.0001. The results are shown that the five models have some predictive value for the differentiation of esophageal squamous cell carcinoma. The five models are constructed as ABC-SVM predictive features. The 10-fold cross-validation accuracy is achieved at 82.0 and 82.5% in the development sets and validation sets, respectively. The new features are constructed by the five models which have a high correlation with the degree of tumor differentiation of esophageal squamous cell carcinoma. And the ABC-SVM algorithm is used to predict the degree of tumor differentiation of esophageal squamous cell carcinoma which can achieve good results.

The main focus of this article is to investigate the indicators significantly associated with the degree of esophageal squamous cell carcinoma differentiation and to develop the model to predict the tumor differentiation of esophageal squamous cell carcinoma. By using Khonen clustering algorithm, ABC-SVM algorithm, logistic regression, ReliefF algorithm, and ROC curve method, the method for predicting the degree of esophageal squamous cell carcinoma differentiation is proposed. The main contributions of this article can be summarized as:

(1) Thirteen indicators associated with the degree of esophageal squamous cell carcinoma differentiation are found in the development sets and are validated in the external validation sets.
(2) Five models with predictive value for esophageal squamous cell carcinoma differentiation are found in the development sets and are validated in the external validation sets.
(3) Based on five prediction models, new features of differentiation degree are constructed and the degree of esophageal squamous cell carcinoma differentiation is well-predicted by ABC-SVM.

The rest of this paper is organized as follows. In section 2, the original data is analyzed and clustered. Thirteen indicators that are significantly correlated with the degree of differentiation are found and validated in section 3. Section 4 provides details of the process of developing and validating five models significantly associated with the degree of esophageal squamous cell carcinoma differentiation. And the five models are constructed as new features and are studied in development sets and validation sets. The conclusions are drawn in section 5.

## 2. Data Set Analysis

### 2.1. Data Introduction

The original dataset for this study contains 211 samples, each with 21 indicators. The 21 indicators include: WBC count, lymphocyte count, monocyte count, neutrophil count, eosinophil count, basophil count, red blood cell count, hemoglobin concentration, platelets count, total protein, albumin, globulin, PT, INR, APTT, TT, FIB, tumor site, tumor length, tumor width, tumor thickness. The gender, age, tumor site information and the population proportions of the original data sets are shown in Table 1. The mean, median, range, and variance information of the 20 indicators in the original sample sets are shown in Table 2.

**Table 1:** The population proportions of the original data sets.

**Project**	**Category**	**Number of population**	**Percentage of population**
Genders	Male Female	135 76	64% 36%
Ages	≥58^*a*^ <58	68 143	32% 68%
Tumor site	Upper chest Middle chest Lower chest	26 139 46	12% 66% 22%

a *Critical threshold for age in the sample sets. Age is used as a variable, and the degree of tumor differentiation is used as a categorical variable. The ROC curve is drawn. After calculating the Youden index, the critical threshold of age for the degree of differentiation is determined to be 58. P < 0.05. The value of AUC is greater than 0.5. The Youden index is decided by (14)*.

*Youden Index = Sensitivity−(1−Specificity) (14)*.

**Table 2:** The original data information.

**Variable**	**Mean**	**Median (Range)**	**Variance**
Tumor length	3.873459716	4 (1.5~10.5)	2.548625592
Tumor width	2.538862559	2.5 (1~7)	0.941911081
Tumor thickness	1.140758294	1 (0.8~5)	0.31937847
WBC count	6.484549763	6 (2.5~15.3)	4.841624915
Lymphocyte count	1.869336493	1.8 (0.4~11.7)	0.849811939
Monocyte count	0.422985782	0.4 (0~1.4)	0.1666239
Neutrophil count	3.8907109	3.4 (0.5~10.6)	3.913749492
Eosinophil count	0.133649289	0.1 (0~0.6)	0.022338524
Basophil count	0.04985782	0 (0~1)	0.007075694
Red blood cell count	5.131516588	4.56 (2.93~5.75)	83.44124436
Hemoglobin concentration	139.2938389	140 (95~100)	250.5799142
Platelets count	235.6729858	226 (100~418)	5328.859219
Total protein	71.32701422	71 (46~92)	57.49731438
Albumin	42.54976303	43 (26~79)	29.04870232
Globulin	28.87203791	28 (17~45)	31.91211916
PT	10.15876777	10.1 (7~16.6)	2.333767998
INR	0.775829384	0.77 (0.45~1.64)	0.027433952
APTT	37.11374408	36.1 (19.7~56.7)	59.97528639
TT	15.45118483	15.6 (8.3~21.3)	4.021748589
FIB	349.3134218	344.029 (245.68~710.56)	4881.231632

*where the unit of tumor length, tumor width, tumor thickness is CM. The unit of WBC count, lymphocyte count, monocyte count, neutrophil count, eosinophil count, basophil count, red blood cell count, hemoglobin concentration, platelets count, total protein, albumin, globulin is g/L. The unit of PT, APTT, TT is second(s). The unit of FIB is mg/L. INR represents the international normalized ratio, which can be expressed by formula 1. ISI is the international sensitivity index for measuring reagents*.

*$INR={\left(\frac{Patient^{\prime }s\;PT}{PT\;of\;normal\;control\;group}\right)}^{ISI}$ (15)*.

### 2.2. Clustering of Data Sets

In order to ensure that the predictive model has some predictive power and application, the method of Khonen clustering is used to cluster all the samples from the original dataset into two different categories of dataset. One type is used as the development sets to develop the prediction model for the degree of esophageal cancer differentiation. Another type is used as the validation sets to validate the developed prediction model.

The Kohonen neural network contains an input layer and a mapping layer. It is able to leverage network architecture to discover features and correlations of data sets (Pastukhov and Prokofiev, 2016). Data with similar characteristics are aggregated and clustered. The Kohonen algorithm is based on the principle of clustering objects with the same characteristics into one class. It not only handles large amounts of multivariate data with high dimensionality, but also preserves the important information implied by the original data (Kumar, 2017; Pasa et al., 2017).

**Figure 1:**
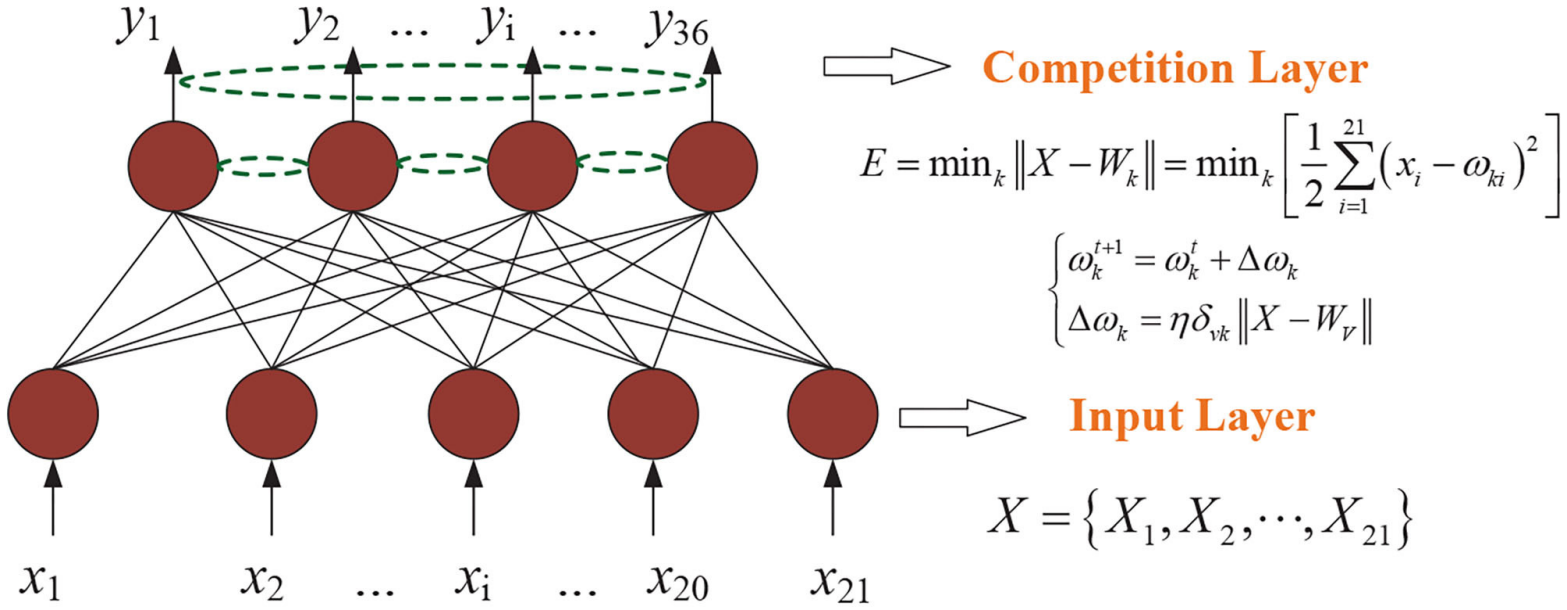
Khonen neural network of 36–21 structure. η is the learning rate. *k* represents the *k*-*th* node of the output layer and ω is regarded as the connection weight value. *X* stands for the initial vector and *i* is the *i*-*th* node of the input layer.

**Figure 2:**
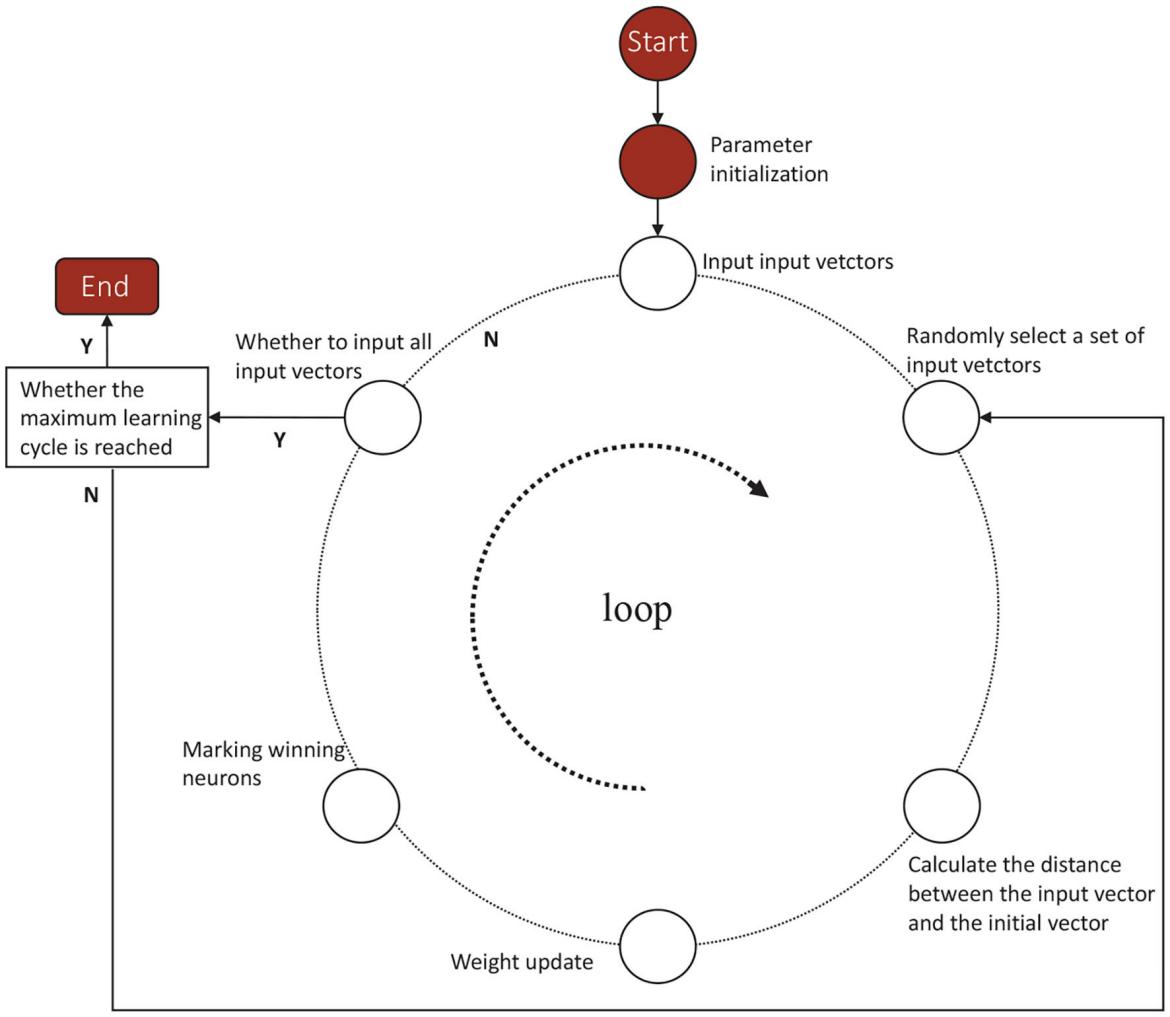
The flow diagram of Kohonen neural network algorithm.

According to the competitive learning algorithm, the connection weights of the winning network's output neurons become stronger and stronger. In order to reduce the distance between the winning neuron and the input vector, the connection weights of neighboring neurons around the winning neuron are adjusted to be closer to the original input vector. Eventually different categories are gradually formed (Sun et al., 2020; Yang et al., 2020). As shown in Figure 1, a Kohonen neural network of 36–21 structure is established. The flow diagram of Kohonen neural network algorithm is shown in Figure 2. Algorithm 1 presents the main procedures of the Khonen neural network algorithm. In the *Kohonen* *algorithm*, *N* is set as 221 and *m* is given as 21. *i* is regarded as 21 and *l* is set as 221.

**Algorithm 1:** Framework of the Khonen neural network algorithm.

**Input:** Clustering indicators *M*. Clustering samples *N*. (*N* = 221, *M* = *M*_1_, *M*_2_, *M*_3_, ...*M*_*m*_, *m* = 21)
**Output:** Relevant indicator *T*. (*T* = *T*_1_, *T*_2_, ..., *T*_*n*_, *n* < *N*)
1: Data normalization (1) $$\begin{array}{l} y=\frac{\left(y_{max}-y_{min}\right)\left(x-x_{min}\right)}{\left(x_{max}-x_{min}\right)}+y_{min} \end{array}$$
2: Randomly set the vector of the initial connection weight value between the mapping layer and the input layer. The initial value η of the learning rate is 0.7, η ∈ (0, 1). The initial neighborhood is set to *N*_*k*0_.
3: Input of initial vector *X* (2) $$\begin{array}{l} X={\left(x_{1},x_{2},x_{3},\cdots \;,x_{m}\right)}^{T},m\in \left[1,221\right] \end{array}$$
4: Calculate the distance between the weight vector of the mapping layer and the initial vector (3) $$\begin{array}{l} X_{i}^{l}=\left(X_{1}^{l},X_{2}^{l},\cdots \;,X_{i}^{l}\right) \end{array}$$ where *i* is the *i*-*th* node of the mapping layer, *i* = 1, 2, ..., 21, *l* is the training data, *l* = 1, 2, ..., 221. *E* is calculated by (4), (4) $$\begin{array}{l} E=\begin{array}{c} \min\\ k \end{array}∥X-W_{k}∥=\begin{array}{c} \min\\ k \end{array}\left[\frac{1}{2}\overset{21}{\underset{i=1}{\sum }}{\left(x_{i}-w_{ki}\right)}^{2}\right] \end{array}$$ where *k* is the *k*-*th* node of the output layer, *k* = 1, 2, ..., 36, *w*_*ki*_ is the connection weight value of the *i*-*th* neuron of the input layer and the *k*-*th* input neuron of the mapping layer.
5: Weight learning (5) $$\begin{array}{l} \{\begin{array}{l} w_{k}^{t+1}=w_{k}^{t}+\Delta w_{k}\\ \Delta w_{k}=\eta \delta_{vk}∥X-W_{v}∥ \end{array} \end{array}$$ where *t* is the number of learning cycles (*t* = 50). ***W***_*v*_ is the weight of the connection between the neurons surrounding the winning neurons and the initial vector. η is a constant of [0, 1], which gradually decreases to 0 by (6), (6) $$\begin{array}{l} \eta \left(t\right)=0.2\left(1-t/1,000\right) \end{array}$$ δ_*vk*_ represents the value of the proximity relationship between the neuron *k* and the adjacent center *v*, as in (7), (7) $$\begin{array}{l} \delta_{vk}=e^{-{\left(D_{vk}/R\right)}^{2}} \end{array}$$ where *D*_*vk*_ represents the distance of the output neuron *k* from the center of the network topology to the adjacent center *v*. *R* is the radius of the winning neighborhood *N*_*kt*_ of neuron *k*.
6: Winning neurons are labeled.
7: End

As shown in Table 3, the original dataset is partitioned into two separate datasets by the Kohonen clustering algorithm. These are two different classes of datasets, one as development sets and one as validation sets. The development sets contain 114 samples, with 53 low differentiation samples and 61 medium differentiation samples. Each sample contains 21 indicators. The validation sets contain 97 samples, with 40 samples for low differentiation and 57 samples for medium differentiation, each sample containing 21 indicators. The development sets is used to find indicators and prediction models that are significantly correlated with the degree of differentiation of esophageal squamous cell carcinoma. For the validation sets, on the one hand, it is used to validate the relevance of the indicators found in the development sets. On the other hand, it is used to validate the validity of the prediction models found in the development sets.

**Table 3:** Numbers of samples in the development sets and validation sets.

**Project**	**Development sets**		**Validation sets**		**Number of samples**
	**Male**	**Female**	**Male**	**Female**	
Poorly differentiated	37	16	28	14	95
Moderate differentiation	36	25	34	21	116
Total number of samples	114		97		211

## 3. Correlation Analysis of Indicators

### 3.1. Clustering of Correlation Indicators

To ensure the rapidity and validity of the predictive model, 21 indicators need to be screened. The Kohonen clustering algorithm is used to screen the indicators that are significantly associated with the differentiation of esophageal squamous cell carcinoma. In the *Kohonen* *algorithm*, *N* is set as 21 and *m* is given as 114. *i* is regarded as 114 and *l* is set as 21.

According to the clustering results, 21 indicators are clustered by Kohonen clustering algorithm in the development sets, and finally 13 indicator that are significantly associated with the degree of esophageal cancer differentiation are found. The 13 indicators that are significantly correlated with the degree of differentiation are WBC count, lymphocyte count, monocyte count, neutrophil count, eosinophil count, basophil count, red blood cell count, PT, INR, tumor site, tumor length, tumor width, tumor thickness in Table 4.

**Table 4:** Information of 13 indicators that are significantly related to the degree of differentiation in the development sets.

**Tumor site**	**Tumor length**	**Tumor width**	**Tumor thickness**	**WBC count**	**Lymphocyte count**	**Monocyte count**	**Neutrophil count**	**Eosinophil count**	**Basophil count**	**ErythVte count**	**PT**	**INR**	**Degree of differentiation**
Lower chest	2.5	3	0.5	7.4	2	0.7	4.3	0.4	0	4.09	10	0.75	Poorly differentiated
Middle chest	3.5	3	0.5	10.4	3.9	0.7	5.6	0.1	0.1	5.02	9.6	0.71	Poorly differentiated
Lower chest	4	1	0.6	6	3.4	0.4	2.2	0	0	4.13	7.1	0.46	Poorly differentiated
Middle chest	4	3	1	7.1	3.5	0.3	3	0.3	0	4.31	11.8	0.95	Moderate differentiation
Upper chest	8	5	1	6.6	1.2	0.6	4.8	0	0	4.24	7	0.45	Moderate differentiation
Middle chest	2	2	1.5	7.7	2.1	0.4	5	0.2	0	4.07	8.8	0.63	Moderate differentiation

### 3.2. Correlation Indicators Validation and ESCC Differentiation Prediction

In recent years, machine learning technology has developed rapidly and has outstanding performance in many fields (Jain et al., 2019; Parikh and Menon, 2019; Sahiner et al., 2019; Sun et al., 2019). Common classification algorithms include Support Vector Machine (SVM) (Hou et al., 2018), Classification And Regression Tree (CART), K-Nearest Neighbor (KNN), Ensemble algorithm, Extreme Learning Machine (ELM), etc. Different algorithms have their own unique advantages. Various classification prediction problems are successfully solved by these algorithms (McCoy and Auret, 2019; Wu and Zhao, 2019).

In order to verify the correlation between these thirteen indicators and the degree of differentiation of esophageal squamous cell carcinoma, the degree of differentiation is predicted based on 21 and 13 indicators, respectively. In this study, 10 different classification algorithms are used to predict the differentiation of esophageal squamous cell carcinoma. Ten classification algorithms used in this paper are SVM (Vadali et al., 2019), Quadratic Discriminant Analysis (QDA) (Siqueira et al., 2017), CART (Cheng et al., 2017), Linear Discriminant Analysis (LDA) (Liu et al., 2016), KNN (Suyundikov et al., 2015), Ensemble (Xiao et al., 2018), ELM (Sachnev et al., 2015), Particle Swarm Optimization-Support Vector Machine (PSO-SVM) (Jiang et al., 2010), Genetic Algorithm-Support Vector Machine (GA-SVM) (Tao et al., 2019), and ABC-SVM (Alshamlan et al., 2016). Thirteen and twenty-one indicators are used as input characteristics, respectively. And the degrees of differentiation of esophageal squamous cell carcinoma are used as the outputs. The average accuracy of the 10-fold cross-validation of 10 classification algorithms are shown in Table 5.

**Table 5:** The prediction results of the degree of differentiation of 10 classification algorithms based on 21 indicators and 13 indicators.

**Data set**	**Number of samples**	**Classification algorithm**	**Number of indicators**	**Training time**	**Average accuracy of 10-fold crossover**
Development sets	114	SVM	21	0.4339	57.9
			13	0.3941	53
		QDA	21	0.5149	57.9
			13	0.3726	57.4
		CART	21	2.7506	54.4
			13	0.3985	59.1
		LDA	21	1.1738	59.6
			13	0.3570	57.4
		KNN	21	0.4219	57.9
			13	0.3879	53.9
		Ensemble	21	3.1797	61.4
			13	3.3533	61.7
		ELM	21	0.0691	58
			13	0.0121	47
		PSO-SVM	21	134.23	58
			13	109.11	59
		GA-SVM	21	8.4211	51
			13	6.7524	50.01
		**ABC-SVM**	21	0.9919	75
			**13**	**0.6273**	**81.5**
Validation sets	97	SVM	21	0.3991	58.8
			13	0.3852	54.8
		QDA	21	0.3802	52.6
			13	0.3642	52.3
		CART	21	0.3444	48.5
			13	0.3609	48.7
		LDA	21	0.3929	49.5
			13	0.3568	61.9
		KNN	21	0.4039	60.8
			13	0.3976	53
		Ensemble	21	3.2612	61.9
			13	3.3079	57.4
		ELM	21	0.0541	53
			13	0.0113	53
		PSO-SVM	21	258.12	60
			13	139.68	58.89
		GA-SVM	21	6.4214	58
			13	4.9353	63
		**ABC-SVM**	21	0.62332	76
			**13**	**0.5006**	**80**

*where SVM is Support Vector Machine and QDA is Quadratic Discriminant Analysis. The CART represents Classification And Regression Tree and the LDA represents Linear Discriminant Analysis. KNN is K-Nearest Neighbor and Ensemble is Ensemble Bagged Tree. The ELM represents Extreme Learning Machine and the PSO-SVM represents Particle Swarm Optimization-Support Vector Machine. GA-SVM is Genetic Algorithm-Support Vector Machine and ABC-SVM is Artificial Bee Colony-Support Vector Machine*.

Cross-validation is used to test the accuracy of the algorithms. Ten-fold cross-validation is a commonly used method to test the classification performance of classifiers. Based on the large datasets, different algorithms are tested and it is shown that 10-fold is an appropriate choice to obtain the best error estimate. The dataset is divided into 10 parts, and nine of them are rotated for training and one for validation. The mean of the 10 times results is used as an estimate of the accuracy of the algorithm.

As shown in Table 5, the degree of differentiation of esophageal squamous cell carcinoma is predicted by 10 different classification algorithms in the development sets and validation sets, based on 21 and 13 indicators, respectively. The results show that ABC-SVM has a higher average accuracy than the other nine algorithms for 10-fold cross-validation and is more efficient in training. In the development sets, the average accuracy of the 10-fold cross-validation of ABC-SVM predicting the degree of differentiation based on 13 indicators is 81.5%. The average accuracy rate based on 21 indicators is only 75.0%. In the validation sets, the average accuracy of the 10-fold cross-validation of ABC-SVM predicting the degree of differentiation based on 13 indicators is 80.0%. The average accuracy rate based on 21 indicators is only 76.0%. Thus, these 13 indicators screened by the clustering algorithm have a higher correlation with the degree of tumor differentiation of esophageal squamous cell carcinoma. The 13 indicators screened by the clustering algorithm not only improved the prediction accuracy of the differentiation degree, but also reduced the training time of the classification algorithm and improved the training efficiency of the classification algorithm.

ABC algorithm is a widely used optimization algorithm. It can solve high dimensional and complex problems, and achieves good results in our study. In order to improve the classification efficiency of the SVM, the SVM parameters *c* and *g* are optimized by ABC. ABC-SVM not only has a few control parameters, but also achieves global optimum in solving complex high-dimensional problems. The ABC-SVM also has some limitations. It has slow running time when solving large sample data. Moreover, SVM is sensitive to the choice of parameters and kernel functions.

## 4. Development and Validation of the Predictive Model for the Degree of Differentiation of Esophageal Squamous Cell Carcinoma

### 4.1. Development of the Predictive Model for the Degree of Differentiation of ESCC

The data from the development sets are analyzed by using the multiple logistic regression approach. A multiple logistic regression model is a linear regression model with multiple independent variables. It is used to reveal the linear relationship between the dependent variable and multiple variables (Linfante et al., 2016; Sun et al., 2017). Its mathematical model can be formulated as

(8) $$\begin{array}{l} Y=\beta_{0}+\beta_{1}x_{1}+\beta_{2}x_{2}+...+\beta_{p}x_{p} \end{array}$$

where *Y* is the dependent variable. β_0_ stands for constant. *x*_1_, *x*_2_, ..., *x*_*p*_ represent independent variables. β_1_, β_2_, ..., β_*p*_ are the weighting coefficients of the corresponding independent variables.

Thirteen indicators significantly associated with the degree of esophageal squamous cell carcinoma differentiation are used as inputs and the degree of differentiation as the output. The resulting model can be expressed as

(9) $$\begin{array}{l} Model\;1=6.59*X_{1}+3.85*X_{2}+4.69*X_{3}+10.005*X_{4}\\ \;+11.74*X_{5}+47.24*X_{6}+5.51*X_{7}+107.13*X_{9}\\ \;+5.571*X_{11}-9.66*X_{12}-13.01*X_{13} \end{array}$$

where *X*_1_ represents the tumor site and *X*_2_ represents the tumor length. *X*_3_ is the tumor width and the *X*_4_ is the tumor thickness. *X*_5_ represents the WBC count and the *X*_6_ represents the lymphocyte count. *X*_7_ is the monocyte count and *X*_9_ is the eosinophil count. *X*_11_ represents the red blood cell count and *X*_12_ represents the PT. *X*_13_ represents the INR.

**Figure 3:**
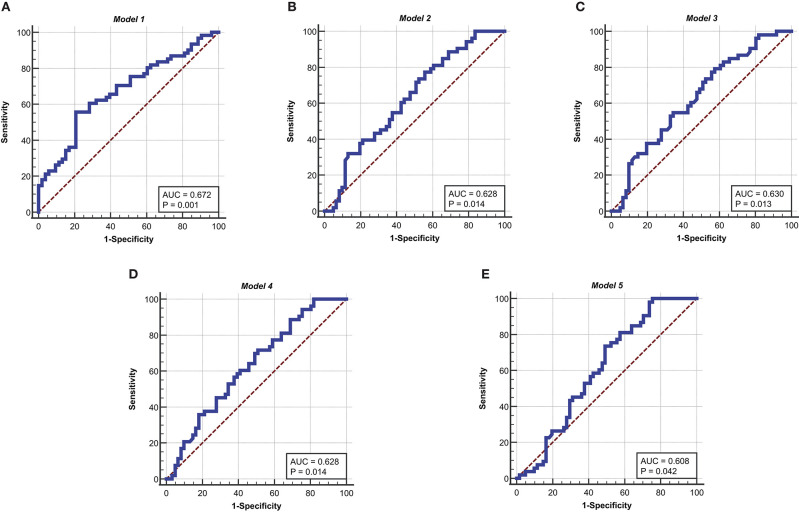
ROC curves for the five models in the development sets. **(A)** ROC curve of *Model* 1. **(B)** ROC curve of *Model* 2. **(C)** ROC curve of *Model* 3. **(D)** ROC curve of *Model* 4. **(E)** ROC curve of *Model* 5. The ordinate is “Sensitivity” and the abscissa is “1-Specificity,” the curves is clearly located at the upper left of the diagonal and has a good significance.

The receiver operating characteristic (ROC) curve is used in a wide range of applications. As a common analytical tool, the ROC curve not only to describe the discrimination accuracy of the prediction model, but also to find critical thresholds for classification (Mas, 2018; Obuchowski and Bullen, 2018; Sun et al., 2018; Luquefernandez et al., 2019). In this study, the ROC curve is used to test the predictive ability of the model. The ROC curve of *Model* 1 in the development sets is shown in Figure 3A. The ROC results for *Model* 1 in the development sets are shown in Table 6. The value of area under curve (AUC) is 0.672, larger than 0.5. *P* = 0.0007. It follows that *Model* 1 has some predictive value for the differentiation of esophageal squamous cell carcinoma.

**Table 6:** Results of ROC curve in the development sets.

**Project**	**Model 1**	**Model 2**	**Model 3**	**Model 4**	**Model 5**
Area under the ROC curve (AUC)	**0.672**	**0.628**	**0.63**	**0.628**	**0.608**
Standard Error	0.0505	0.0522	0.0522	0.0521	0.0532
95% Condence interval	0.578 to 0.757	0.533 to 0.717	0.534 to 0.718	0.533 to 0.717	0.512 to 0.698
Z statistic	3.406	2.452	2.483	2.459	2.037
Significance level P (Area = 0.5)	**0.0007**	**0.0142**	**0.013**	**0.014**	**0.0417**
Youden index J	0.3498	0.2162	0.2187	0.2088	0.2459
Associated criterion	>-17.25294	>0.98361	>-0.79807	>0.732	>1.9012
Sensitivity	79.25	77.36	79.25	71.7	100
Specificity	55.74	44.26	42.62	49.18	24.59

Then, the Kohonen algorithm is used again to obtain 5 indicators that are significantly related to the degree of differentiation. In the *Kohonen* *algorithm*, *N* is set as 13 and *m* is given as 114. *i* is regarded as 114 and *l* is set as 13. Thirteen indicators are clustered by Kohonen algorithm to obtain five relevant indicators, which are tumor thickness, monocyte count, eosinophil count, basophil count, and INR. Multiple logistic regression is used, with five correlation indicators as inputs and the degrees of differentiation as outputs. The *Model* 2 and the *Model* 3 are obtained. The *Model* 2 can be expressed by Equation (10). The *Model* 3 can be represented by Equation (11). The ROC curve of *Model* 2 and *Model* 3 in the development sets is shown in Figures 3B,C, respectively. The ROC results for *Model* 2 and *Model* 3 in the development sets are shown in Table 6. The value of area under curve (AUC) for *Model* 2 is 0.628, larger than 0.5. *P* = 0.0142. The value of area under curve (AUC) for *Model* 3 is 0.630, larger than 0.5. *P* = 0.013. The results show that *Model* 2 and *Model* 3 have good predictive value for the differentiation of esophageal squamous cell carcinoma.

(10) $$\begin{array}{l} \begin{array}{l} Model\;2=5.85*X_{1}+3.66*X_{2}+38.66*X_{3}\\ \;+8.32*X_{4}-12.38*X_{5} \end{array} \end{array}$$

(11) $$\begin{array}{l} \begin{array}{l} Model\;3=5.85*X_{1}+38.67*X_{3}+8.32*X_{4}\\ \;-12.38*X_{5} \end{array} \end{array}$$

where *X*_1_ is the tumor thickness and *X*_2_ is the monocyte count. *X*_3_ represents the eosinophil count and *X*_4_ represents the basophil count. *X*_5_ is the INR.

ReliefF is a feature weighting algorithm and runs efficiently. It has no restriction on the data type and assigns higher weight to all features that are highly correlated with the category (Palmamendoza et al., 2018; Urbanowicz et al., 2018). Algorithm 2 presents the main procedures of the ReliefF algorithm.

**Algorithm 2:** Framework of ReliefF algorithm.

**Input:** *N* indicators related to the degree of differentiation. The degree of differentiation corresponding to the sample *M*. (*N* = 5, *M* stands for the degree of differentiation, including low differentiation and medium differentiation)
**Output:** Feature weight of each indicator: *T*. (*T* = {*W*_1_, *W*_2_, *W*_3_, *W*_4_, *W*_5_})
1: Set training data as *D* and sample sampling times as *m*. δ is represents the feature weight threshold. Set the number of nearest neighbor samples as *k* (*D* = 114, *m* = 80, *k* = 8);
2: for *i* = 1 to *m* *do*
3: Randomly select a sample *R* from *D*
4: In the same sample sets of *R*, find the *k* nearest neighbors *H*_*j*_(*j* = 1, 2, ..., *k*) of *R*. In the different sample sets of *R*, find the *k* nearest neighbors *M*_*j*_(*C*) of *R*;
5: for *A* = 1 to *N* All indicators *do*
6: $W\left(A\right)=W\left(A\right)-\overset{k}{\underset{j=1}{\sum }}diff\left(A,R,H_{j}\right)/\left(mk\right)$
7: +$\underset{Ceclass\left(R\right)}{\sum }\left[\frac{p\left(C\right)}{1-P\left(Class\left(R\right)\right)}\overset{k}{\underset{j=1}{\sum }}diff\left(A,R,M_{j}\left(C\right)\right)\right]/\left(mk\right)$
8: end

Five indicators significantly associated with esophageal squamous cell carcinoma are used as inputs and the degrees of differentiation as outputs. *Model* 4 and *Model* 5 are obtained by the ReliefF algorithm. The *Model* 4 is decided by (12). The *Model* 5 could be written as (13). The ROC curve of *Model* 4 and *Model* 5 in the development sets are shown in Figures 3D,E, respectively. The ROC results for *Model* 4 and *Model* 5 in the development sets are shown in Table 6. The value of area under curve (AUC) for *Model* 4 is 0.628, larger than 0.5. *P* = 0.0142. The value of area under curve (AUC) for *Model* 5 is 0.608, larger than 0.5. *P* = 0.0417. Therefore, *Model* 4 and *Model* 5 have some predictive value for the differentiation of esophageal squamous cell carcinoma.

(12) $$\begin{array}{l} \begin{array}{l} Model\;4=1.08*X_{1}+1.5*X_{2}+2.09*X_{3}\\ \;+0.56*X_{4}-1.5*X_{5} \end{array} \end{array}$$

(13) $$\begin{array}{l} \begin{array}{l} Model\;5=1.7*X_{1}+1.5*X_{2}-0.28*X_{3}\\ \;-0.92*X_{4}+0.84*X_{5} \end{array} \end{array}$$

where *X*_1_ is the tumor thickness and *X*_2_ is the monocyte count. *X*_3_ represents the eosinophil count and *X*_4_ represents the basophil count. *X*_5_ is the INR.

### 4.2. Validation of the Predictive Model for the Degree of Differentiation of ESCC

**Figure 4:**
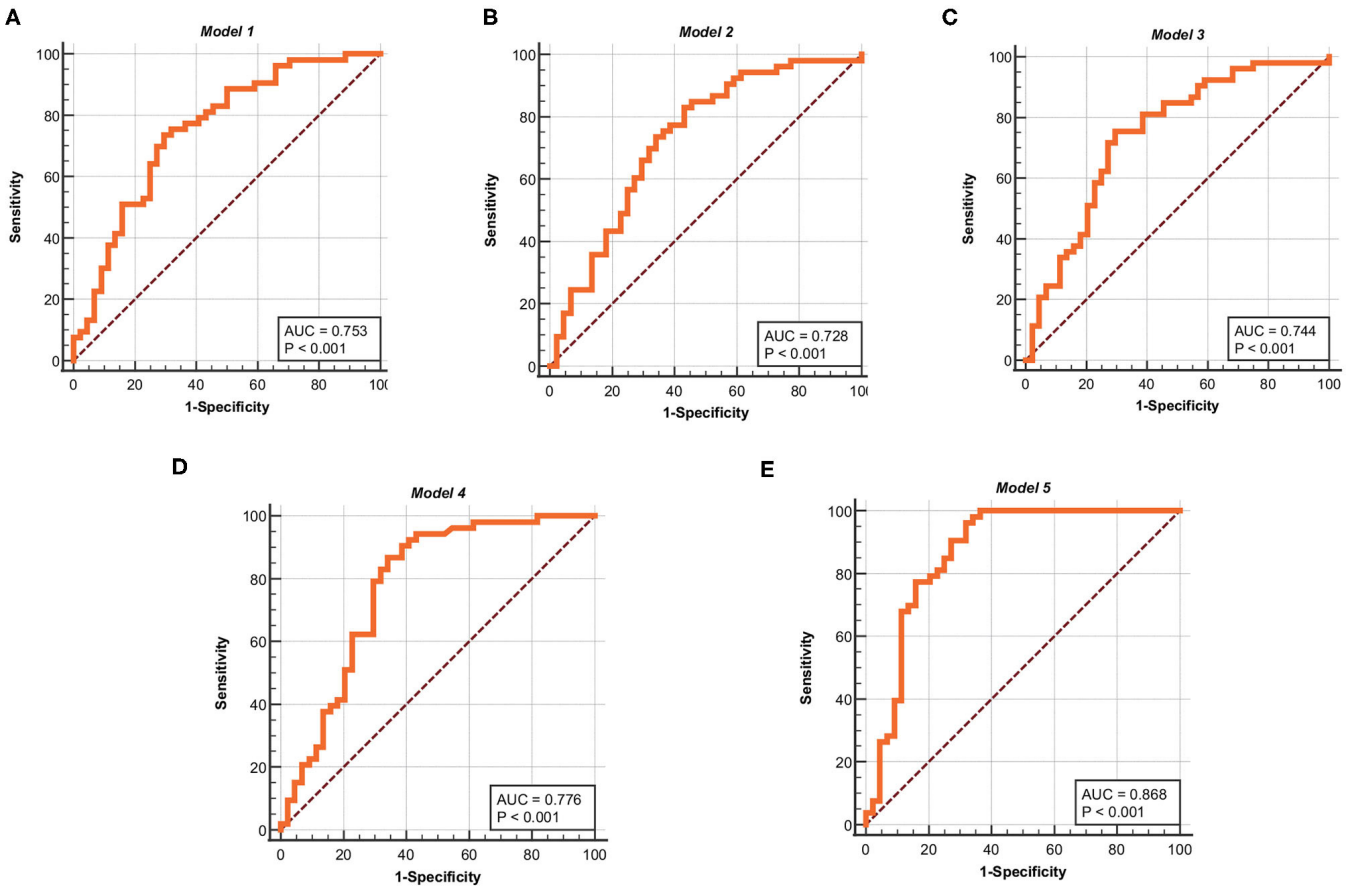
ROC curves for the five models in the validation sets. **(A)** ROC curve of *Model* 1. **(B)** ROC curve of *Model* 2. **(C)** ROC curve of *Model* 3. **(D)** ROC curve of *Model* 4. **(E)** ROC curve of *Model* 5. The ordinate is “Sensitivity” and the abscissa is “1-Specificity,” the curves is clearly located at the upper left of the diagonal and has a good significance.

In this paper, in order to test the validity of the models, the five models obtained in the development sets are used for testing and evaluation in the validation sets. The ROC curves of *Model* 1, *Model* 2, *Model* 3, *Model* 4, and *Model* 5 are shown in Figures 4A–E, respectively. The ROC analysis of the five models in the validation sets are shown in Table 7. The results show that the five models in the validation sets also have good predictive value. The AUC values of the five models in the validation sets are 0.753, 0.728, 0.744, 0.776, and 0.868. The *P*-values for five models are less than 0.0001. Therefore, the five models have some application performance and potential predictive capability.

**Table 7:** Results of ROC curve in the validation sets.

**Project**	**Model 1**	**Model 2**	**Model 3**	**Model 4**	**Model 5**
Area under the ROC curve (AUC)	**0.753**	**0.728**	**0.744**	**0.776**	**0.868**
Standard Error	0.0505	0.053	0.0517	0.0507	0.0409
95% Condence interval	0.655 to 0.835	0.628 to 0.814	0.645 to 0.827	0.680 to 0.855	0.784 to 0.928
Z statistic	4.999	4.307	4.716	5.448	8.995
Significance level P (Area = 0.5)	**<0.0001**	**<0.0001**	**<0.0001**	**<0.0001**	**<0.0001**
Youden index J	0.4404	0.3984	0.4593	0.527	0.6441
Associated criterion	≤ −12.37098	≤ 3.96107	≤ 0.92197	≤ 1.074	≤ 3.4248
Sensitivity	73.58	83.02	75.47	86.79	96.23
Specificity	70.45	56.82	70.45	65.91	68.18

### 4.3. Constructing New Features to Predict the Degree of Differentiation of ESCC

To better achieve the accurate prediction of the degree of esophageal squamous cell carcinoma differentiation, new features are constructed based on the five models obtained in this paper. The predict values of the five models are taken as input features and the degrees of differentiation as the outputs. The ABC-SVM algorithm is used to predict the degree of differentiation of esophageal squamous cell carcinoma. In the development sets, the average accuracy of the 10-fold cross-validation of the ABC-SVM based on the five models features is 82%. The average accuracy of the 10-fold cross-validation of the ABC-SVM based on 21 and 13 indicators is only 75 and 81.5%, respectively. In the validation sets, the average accuracy of the 10-fold cross-validation of the ABC-SVM model based on the five model features is 86.5%. The average accuracy of the 10-fold cross-validation of the ABC-SVM based on 21 and 13 indicators is only 76 and 80%, respectively. As shown in Table 8, the prediction accuracy is improved by combining the features of the five models. It reached 82 and 86.5% in the development sets and validation sets, respectively. Based on the new features of the five model constructs, the operational efficiency of the ABC-SVM is enhanced, and the prediction accuracy of esophageal squamous cell carcinoma differentiation is effectively improved.

**Table 8:** ABC-SVM prediction results based on the new features of the five model constructs.

**Project**	**Number of samples**	**Number of indicators**	**Average accuracy of 10-fold crossover**
Development sets	114	21 indicators	75%
		13 indicators	81.5%
		**Five models as features**	**82%**
Validation sets	97	21 indicators	76%
		13 indicators	80%
		**Five models as features**	**86.5%**

## 5. Conclusions

In this paper, the Kokonen clustering algorithm, ABC-SVM, logistic regression, ReliefF, and ROC are used to analyze and predict the tumor differentiation of esophageal squamous cell carcinoma. Thirteen indicators significantly associated with esophageal squamous cell carcinoma are found in the development sets by the Kohonen clustering algorithm. Ten classification algorithms are used to predict the tumor differentiation of esophageal squamous cell carcinoma based on 13 significantly correlated indicators. The results showed that ABC-SVM have a good prediction performance, with the 10-fold cross-validation accuracy of 81.5%. Five models with high predictive value for esophageal squamous cell carcinoma differentiation are found in the development sets by the logistic regression and ReliefF algorithm. The AUC values of the five models in the development sets are 0.672, 0.628, 0.630, 0.628, and 0.608 (*P* < 0.05). The AUC values of the five models in the validation set are 0.753, 0.728, 0.744, 0.776, and 0.868 (*P* < 0.001). The five models are used as input features and the ABC-SVM algorithm is used to predict the degree of tumor differentiation. The 10-fold cross-validation accuracy of ABC-SVM in the development sets and validation sets is 82.0 and 86.5%, respectively. In this study, tumor differentiation of esophageal squamous cell carcinoma patients is effectively analyzed and predicted, which can assist physicians in their diagnostic decisions and provide timely diagnosis and effective treatment of patients.

## Data Availability Statement

The datasets presented in this article are not readily available because the data used in the study are private and confidential data. Requests to access the datasets should be directed to Junwei Sun, junweisun@yeah.net.

## Ethics Statement

Ethical review and approval was not required for the study on human participants in accordance with the local legislation and institutional requirements. The ethics committee waived the requirement of written informed consent for participation.

## Author Contributions

All authors listed have made a substantial, direct and intellectual contribution to the work, and approved it for publication.

## Conflict of Interest

The authors declare that the research was conducted in the absence of any commercial or financial relationships that could be construed as a potential conflict of interest.
